# Supplementary material for: Classifying Breast Cancer Subtypes Using Multiple Kernel Learning Based on Omics Data
Source: Genes (Basel). 2019 Mar 7;10(3):200. doi: 10.3390/genes10030200 (PMC6471546; doi:10.3390/genes10030200)
Supplement: Supplementary file 1 [file genes-10-00200-s001.zip › Table S1. The precision of multi-classification in breast cancer subtypes .docx]

Table S1. The precision of multi-classification in breast cancer subtypes

| Breast cancer subtypes | RNA | Methylation | CNV | MKL |
| --- | --- | --- | --- | --- |
| Luminal A | 0.625 | 0.644 | 0.487 | **0.676** |
| Luminal B | 0 | 0 | **0.667** | 0.333 |
| TNBC | 0.634 | 0.618 | 0.387 | **0.641** |
| HER2 (+) | 0 | 0 | 0 | **0.25** |
| Unclear | 0.808 | 0.669 | 0.371 | **0.846** |
