# Supplementary material for: Classifying Breast Cancer Subtypes Using Multiple Kernel Learning Based on Omics Data
Source: Genes (Basel). 2019 Mar 7;10(3):200. doi: 10.3390/genes10030200 (PMC6471546; doi:10.3390/genes10030200)
Supplement: Supplementary file 1 [file genes-10-00200-s001.zip › Table S2. The recall of multi-classification in breast cancer subtypes.docx]

Table S2. The recall of multi-classification in breast cancer subtypes

| Breast cancer subtypes | RNA | Methylation | CNV | MKL |
| --- | --- | --- | --- | --- |
| Luminal A | 0.874 | 0.820 | 0.658 | **0.906** |
| Luminal B | 0 | 0 | **0.08** | 0.06 |
| TNBC | **0.658** | 0.595 | 0.152 | 0.633 |
| HER2 (+) | 0 | 0 | 0 | **0.1** |
| Unclear | 0.641 | 0.645 | 0.392 | **0.682** |
