# Supplementary material for: Classifying Breast Cancer Subtypes Using Multiple Kernel Learning Based on Omics Data
Source: Genes (Basel). 2019 Mar 7;10(3):200. doi: 10.3390/genes10030200 (PMC6471546; doi:10.3390/genes10030200)
Supplement: Supplementary file 1 [file genes-10-00200-s001.zip › Table S3. The top 30 pathways in these classification on expression data.docx]

Table S3. The top 30 pathways in these classification on expression data

| **Classification** | **P-value** | **PATHWAY** | |  |
| --- | --- | --- | --- | --- |
| **Luminal A**  **versus**  **Luminal B** | 0 | KEGG_CELL_CYCLE | |  |
|  | 0 | REACTOME_CELL_CYCLE | |  |
|  | 0 | REACTOME_ORC1_REMOVAL_FROM_CHROMATIN | |  |
|  | 0 | REACTOME_CELL_CYCLE_MITOTIC | |  |
|  | 0 | REACTOME_CELL_CYCLE_CHECKPOINTS | |  |
|  | 0 | REACTOME_M_G1_TRANSITION | |  |
|  | 0 | REACTOME_G1_S_TRANSITION | |  |
|  | 0 | REACTOME_SYNTHESIS_OF_DNA | |  |
|  | 0 | REACTOME_MITOTIC_G1_G1_S_PHASES | |  |
|  | 0 | REACTOME_REGULATION_OF_MITOTIC_CELL_CYCLE | |  |
|  | 0 | REACTOME_MITOTIC_M_M_G1_PHASES | |  |
|  | 0 | REACTOME_ASSEMBLY_OF_THE_PRE_REPLICATIVE_COMPLEX | |  |
|  | 0 | REACTOME_DNA_REPLICATION | |  |
|  | 0 | REACTOME_HIV_INFECTION | |  |
|  | 0 | REACTOME_MITOTIC_PROMETAPHASE | |  |
|  | 0 | REACTOME_IMMUNE_SYSTEM | |  |
|  | 0 | REACTOME_ADAPTIVE_IMMUNE_SYSTEM | |  |
|  | 0 | REACTOME_G2_M_CHECKPOINTS | |  |
|  | 0 | REACTOME_S_PHASE | |  |
|  | 0.000000000000000222044604925031 | REACTOME_HOST_INTERACTIONS_OF_HIV_FACTORS | |  |
|  | 0.00000000000000199840144432528 | REACTOME_APOPTOSIS | |  |
|  | 0.00000000000000277555756156289 | KEGG_OOCYTE_MEIOSIS | |  |
|  | 0.00000000000000788258347483861 | REACTOME_APC_C_CDC20_MEDIATED_DEGRADATION_OF_MITOTIC_PROTEINS | |  |
|  | 0.00000000000000965894031423886 | REACTOME_REGULATION_OF_MRNA_STABILITY_BY_PROTEINS_THAT_BIND_AU_RICH_ELEMENTS | |  |
|  | 0.000000000000010325074129014 | REACTOME_ACTIVATION_OF_ATR_IN_RESPONSE_TO_REPLICATION_STRESS | |  |
|  | 0.0000000000000118793863634892 | REACTOME_CYCLIN_E_ASSOCIATED_EVENTS_DURING_G1_S_TRANSITION_ | |  |
|  | 0.0000000000000122124532708767 | REACTOME_METABOLISM_OF_LIPIDS_AND_LIPOPROTEINS | |  |
|  | 0.0000000000000133226762955019 | REACTOME_P53_INDEPENDENT_G1_S_DNA_DAMAGE_CHECKPOINT | |  |
|  | 0.0000000000000217603712826531 | REACTOME_ACTIVATION_OF_THE_PRE_REPLICATIVE_COMPLEX | |  |
|  | 0.0000000000000217603712826531 | REACTOME_DNA_STRAND_ELONGATION | |  |
| **Luminal A**  **Versus**  **HER2 (+)** | 0 | | REACTOME_CELL_CYCLE |  |
|  | 0 | | REACTOME_GENERIC_TRANSCRIPTION_PATHWAY |  |
|  | 0 | | REACTOME_CELL_CYCLE_MITOTIC |  |
|  | 0 | | REACTOME_CELL_CYCLE_CHECKPOINTS |  |
|  | 0 | | REACTOME_CYCLIN_E_ASSOCIATED_EVENTS_DURING_G1_S_TRANSITION_ |  |
|  | 0 | | REACTOME_P53_DEPENDENT_G1_DNA_DAMAGE_RESPONSE |  |
|  | 0 | | REACTOME_M_G1_TRANSITION |  |
|  | 0 | | REACTOME_G1_S_TRANSITION |  |
|  | 0 | | REACTOME_SYNTHESIS_OF_DNA |  |
|  | 0 | | REACTOME_MITOTIC_G1_G1_S_PHASES |  |
|  | 0 | | REACTOME_REGULATION_OF_MITOTIC_CELL_CYCLE |  |
|  | 0 | | REACTOME_MITOTIC_M_M_G1_PHASES |  |
|  | 0 | | REACTOME_ASSEMBLY_OF_THE_PRE_REPLICATIVE_COMPLEX |  |
|  | 0 | | REACTOME_DNA_REPLICATION |  |
|  | 0 | | REACTOME_SCF_BETA_TRCP_MEDIATED_DEGRADATION_OF_EMI1 |  |
|  | 0 | | REACTOME_IMMUNE_SYSTEM |  |
|  | 0 | | REACTOME_S_PHASE |  |
|  | 0 | | REACTOME_SCFSKP2_MEDIATED_DEGRADATION_OF_P27_P21 |  |
|  | 0.000000000000000111022302462516 | | REACTOME_SIGNALING_BY_WNT |  |
|  | 0.000000000000000111022302462516 | | REACTOME_P53_INDEPENDENT_G1_S_DNA_DAMAGE_CHECKPOINT |  |
|  | 0.000000000000000222044604925031 | | REACTOME_APC_C_CDH1_MEDIATED_DEGRADATION_OF_CDC20_AND_OTHER_APC_C_CDH1_TARGETED_PROTEINS_IN_LATE_MITOSIS_EARLY_G1 |  |
|  | 0.000000000000000333066907387547 | | REACTOME_ORC1_REMOVAL_FROM_CHROMATIN |  |
|  | 0.000000000000000333066907387547 | | REACTOME_APC_C_CDC20_MEDIATED_DEGRADATION_OF_MITOTIC_PROTEINS |  |
|  | 0.000000000000000444089209850063 | | REACTOME_DESTABILIZATION_OF_MRNA_BY_AUF1_HNRNP_D0 |  |
|  | 0.00000000000000077715611723761 | | REACTOME_METABOLISM_OF_AMINO_ACIDS_AND_DERIVATIVES |  |
|  | 0.000000000000000999200722162641 | | REACTOME_ER_PHAGOSOME_PATHWAY |  |
|  | 0.00000000000000111022302462516 | | REACTOME_REGULATION_OF_ORNITHINE_DECARBOXYLASE_ODC |  |
|  | 0.00000000000000188737914186277 | | REACTOME_ANTIGEN_PROCESSING_UBIQUITINATION_PROTEASOME_DEGRADATION |  |
|  | 0.00000000000000233146835171283 | | REACTOME_CDK_MEDIATED_PHOSPHORYLATION_AND_REMOVAL_OF_CDC6 |  |
|  | 0.00000000000000321964677141295 | | REACTOME_SIGNALING_BY_THE_B_CELL_RECEPTOR_BCR |  |
| **Luminal A**  **versus**  **TNBC** | 0 | | KEGG_CELL_CYCLE |  |
|  | 0 | | KEGG_UBIQUITIN_MEDIATED_PROTEOLYSIS |  |
|  | 0 | | KEGG_ENDOCYTOSIS |  |
|  | 0 | | KEGG_HUNTINGTONS_DISEASE |  |
|  | 0 | | KEGG_PATHWAYS_IN_CANCER |  |
|  | 0 | | REACTOME_GAP_JUNCTION_DEGRADATION |  |
|  | 0 | | REACTOME_SIGNALING_BY_RHO_GTPASES |  |
|  | 0 | | REACTOME_SIGNALLING_BY_NGF |  |
|  | 0 | | REACTOME_DEVELOPMENTAL_BIOLOGY |  |
|  | 0 | | REACTOME_ANTIGEN_PROCESSING_CROSS_PRESENTATION |  |
|  | 0 | | REACTOME_CELL_CYCLE |  |
|  | 0 | | REACTOME_MEMBRANE_BINDING_AND_TARGETTING_OF_GAG_PROTEINS |  |
|  | 0 | | REACTOME_DOWNSTREAM_SIGNALING_EVENTS_OF_B_CELL_RECEPTOR_BCR |  |
|  | 0 | | REACTOME_ACTIVATION_OF_NF_KAPPAB_IN_B_CELLS |  |
|  | 0 | | REACTOME_SIGNALING_BY_THE_B_CELL_RECEPTOR_BCR |  |
|  | 0 | | REACTOME_PROCESSING_OF_CAPPED_INTRON_CONTAINING_PRE_MRNA |  |
|  | 0 | | REACTOME_GENERIC_TRANSCRIPTION_PATHWAY |  |
|  | 0 | | REACTOME_CELL_CYCLE_MITOTIC |  |
|  | 0 | | REACTOME_CELL_CYCLE_CHECKPOINTS |  |
|  | 0 | | REACTOME_CYCLIN_E_ASSOCIATED_EVENTS_DURING_G1_S_TRANSITION_ |  |
|  | 0 | | REACTOME_P53_DEPENDENT_G1_DNA_DAMAGE_RESPONSE |  |
|  | 0 | | REACTOME_MRNA_PROCESSING |  |
|  | 0 | | REACTOME_METABOLISM_OF_PROTEINS |  |
|  | 0 | | REACTOME_M_G1_TRANSITION |  |
|  | 0 | | REACTOME_MRNA_SPLICING |  |
|  | 0 | | REACTOME_G1_S_TRANSITION |  |
|  | 0 | | REACTOME_AXON_GUIDANCE |  |
|  | 0 | | REACTOME_SYNTHESIS_OF_DNA |  |
|  | 0 | | REACTOME_METABOLISM_OF_MRNA |  |
|  | 0 | | REACTOME_METABOLISM_OF_RNA |  |
| **Luminal B**  **Versus**  **HER2 (+)** | 0.00000000000365352192943647 | | REACTOME_GENERIC_TRANSCRIPTION_PATHWAY |  |
|  | 0.00000000318155501943806 | | REACTOME_SIGNALING_BY_NOTCH |  |
|  | 0.00000000572619651695305 | | REACTOME_SIGNALING_BY_NOTCH1 |  |
|  | 0.0000000138016432726928 | | REACTOME_METABOLISM_OF_LIPIDS_AND_LIPOPROTEINS |  |
|  | 0.000000069556717163799 | | KEGG_PATHWAYS_IN_CANCER |  |
|  | 0.000000165510408378644 | | KEGG_PROSTATE_CANCER |  |
|  | 0.000000345059765982647 | | REACTOME_FATTY_ACID_TRIACYLGLYCEROL_AND_KETONE_BODY_METABOLISM |  |
|  | 0.000000374175476491878 | | REACTOME_NOTCH1_INTRACELLULAR_DOMAIN_REGULATES_TRANSCRIPTION |  |
|  | 0.000000401665657290273 | | REACTOME_DEVELOPMENTAL_BIOLOGY |  |
|  | 0.000000606122890656202 | | REACTOME_SIGNALING_BY_THE_B_CELL_RECEPTOR_BCR |  |
|  | 0.00000113549410274416 | | REACTOME_METABOLISM_OF_AMINO_ACIDS_AND_DERIVATIVES |  |
|  | 0.00000130954026600349 | | REACTOME_DOWNSTREAM_SIGNALING_EVENTS_OF_B_CELL_RECEPTOR_BCR |  |
|  | 0.000001387611392345 | | KEGG_UBIQUITIN_MEDIATED_PROTEOLYSIS |  |
|  | 0.00000230923726440846 | | REACTOME_SIGNALLING_BY_NGF |  |
|  | 0.00000300021800136996 | | REACTOME_SIGNALING_BY_INSULIN_RECEPTOR |  |
|  | 0.00000698927099129776 | | KEGG_INSULIN_SIGNALING_PATHWAY |  |
|  | 0.00000838026850347173 | | REACTOME_SIGNALING_BY_WNT |  |
|  | 0.00000913163256632821 | | REACTOME_SIGNALING_BY_ERBB4 |  |
|  | 0.00000952566855672021 | | REACTOME_PPARA_ACTIVATES_GENE_EXPRESSION |  |
|  | 0.0000113401939867419 | | KEGG_ACUTE_MYELOID_LEUKEMIA |  |
|  | 0.0000122276382669151 | | REACTOME_ADAPTIVE_IMMUNE_SYSTEM |  |
|  | 0.0000123789970234434 | | REACTOME_IMMUNE_SYSTEM |  |
|  | 0.0000124626551011309 | | REACTOME_ANTIGEN_PROCESSING_UBIQUITINATION_PROTEASOME_DEGRADATION |  |
|  | 0.0000131624521929607 | | KEGG_CHRONIC_MYELOID_LEUKEMIA |  |
|  | 0.0000147093216668059 | | KEGG_ENDOCYTOSIS |  |
|  | 0.0000153551355762671 | | KEGG_GLYCINE_SERINE_AND_THREONINE_METABOLISM |  |
|  | 0.0000186062683209531 | | REACTOME_METABOLISM_OF_POLYAMINES |  |
|  | 0.0000213352029663216 | | REACTOME_METABOLISM_OF_CARBOHYDRATES |  |
|  | 0.0000213352029663216 | | REACTOME_CLASS_I_MHC_MEDIATED_ANTIGEN_PROCESSING_PRESENTATION |  |
|  | 0.0000217718918540388 | | REACTOME_INSULIN_RECEPTOR_SIGNALLING_CASCADE |  |
| **Luminal B**  **Versus**  **TNBC** | 0 | | KEGG_CELL_CYCLE |  |
|  | 0 | | KEGG_PATHWAYS_IN_CANCER |  |
|  | 0 | | REACTOME_SIGNALLING_BY_NGF |  |
|  | 0 | | REACTOME_DEVELOPMENTAL_BIOLOGY |  |
|  | 0 | | REACTOME_CELL_CYCLE |  |
|  | 0 | | REACTOME_CELL_CYCLE_MITOTIC |  |
|  | 0 | | REACTOME_METABOLISM_OF_PROTEINS |  |
|  | 0 | | REACTOME_METABOLISM_OF_MRNA |  |
|  | 0 | | REACTOME_METABOLISM_OF_RNA |  |
|  | 0 | | REACTOME_MITOTIC_G1_G1_S_PHASES |  |
|  | 0 | | REACTOME_MITOTIC_M_M_G1_PHASES |  |
|  | 0 | | REACTOME_METABOLISM_OF_LIPIDS_AND_LIPOPROTEINS |  |
|  | 0 | | REACTOME_FATTY_ACID_TRIACYLGLYCEROL_AND_KETONE_BODY_METABOLISM |  |
|  | 0 | | REACTOME_DNA_REPLICATION |  |
|  | 0 | | REACTOME_IMMUNE_SYSTEM |  |
|  | 0 | | REACTOME_ADAPTIVE_IMMUNE_SYSTEM |  |
|  | 0.000000000000000222044604925031 | | REACTOME_GENERIC_TRANSCRIPTION_PATHWAY |  |
|  | 0.000000000000000555111512312578 | | REACTOME_SIGNALING_BY_NOTCH |  |
|  | 0.00000000000000177635683940025 | | REACTOME_HIV_INFECTION |  |
|  | 0.00000000000000333066907387547 | | REACTOME_CLASS_I_MHC_MEDIATED_ANTIGEN_PROCESSING_PRESENTATION |  |
|  | 0.00000000000000466293670342566 | | KEGG_ENDOCYTOSIS |  |
|  | 0.00000000000000521804821573824 | | REACTOME_G1_S_TRANSITION |  |
|  | 0.00000000000000643929354282591 | | REACTOME_TOLL_RECEPTOR_CASCADES |  |
|  | 0.00000000000000721644966006352 | | REACTOME_G1_PHASE |  |
|  | 0.00000000000000888178419700125 | | REACTOME_SIGNALING_BY_RHO_GTPASES |  |
|  | 0.0000000000000155431223447522 | | REACTOME_POST_TRANSLATIONAL_PROTEIN_MODIFICATION |  |
|  | 0.0000000000000187627691161651 | | KEGG_MAPK_SIGNALING_PATHWAY |  |
|  | 0.0000000000000240918396343659 | | REACTOME_APOPTOSIS |  |
|  | 0.0000000000000240918396343659 | | REACTOME_S_PHASE |  |
|  | 0.0000000000000259792187762287 | | REACTOME_MEMBRANE_TRAFFICKING |  |
| **HER2 (+)**  **Versus**  **TNBC** | 0.0000000000000185407245112401 | | REACTOME_METABOLISM_OF_LIPIDS_AND_LIPOPROTEINS | |
|  | 0.000000000000287103674168065 | | REACTOME_METABOLISM_OF_PROTEINS | |
|  | 0.00000000000096034291630076 | | REACTOME_METABOLISM_OF_RNA | |
|  | 0.00000000000199507077525141 | | REACTOME_CELL_CYCLE_MITOTIC | |
|  | 0.00000000000694477808593774 | | REACTOME_METABOLISM_OF_MRNA | |
|  | 0.0000000000286416446115823 | | KEGG_PATHWAYS_IN_CANCER | |
|  | 0.0000000000884040618487347 | | REACTOME_INFLUENZA_LIFE_CYCLE | |
|  | 0.000000000357595397737498 | | REACTOME_CELL_CYCLE | |
|  | 0.00000000114787723770604 | | REACTOME_INFLUENZA_VIRAL_RNA_TRANSCRIPTION_AND_REPLICATION | |
|  | 0.00000000136257827243469 | | REACTOME_NONSENSE_MEDIATED_DECAY_ENHANCED_BY_THE_EXON_JUNCTION_COMPLEX | |
|  | 0.00000000178075487689 | | REACTOME_SIGNALING_BY_EGFR_IN_CANCER | |
|  | 0.00000000225174090484614 | | KEGG_MAPK_SIGNALING_PATHWAY | |
|  | 0.00000000444951930855808 | | REACTOME_3_UTR_MEDIATED_TRANSLATIONAL_REGULATION | |
|  | 0.0000000048866363178135 | | KEGG_SPLICEOSOME | |
|  | 0.00000000490380858142458 | | REACTOME_FATTY_ACID_TRIACYLGLYCEROL_AND_KETONE_BODY_METABOLISM | |
|  | 0.00000000602120220349889 | | REACTOME_ADAPTIVE_IMMUNE_SYSTEM | |
|  | 0.00000000609849382104954 | | REACTOME_DNA_REPLICATION | |
|  | 0.00000000838420433080245 | | REACTOME_IMMUNE_SYSTEM | |
|  | 0.0000000110044653389707 | | REACTOME_METABOLISM_OF_VITAMINS_AND_COFACTORS | |
|  | 0.0000000147387066995464 | | REACTOME_TRANSLATION | |
|  | 0.0000000285742585148796 | | REACTOME_DEVELOPMENTAL_BIOLOGY | |
|  | 0.0000000307844820746084 | | REACTOME_PEPTIDE_CHAIN_ELONGATION | |
|  | 0.0000000418331688178952 | | REACTOME_MITOTIC_G1_G1_S_PHASES | |
|  | 0.0000000531010417859079 | | KEGG_RIBOSOME | |
|  | 0.0000000562187448815976 | | REACTOME_CLASS_I_MHC_MEDIATED_ANTIGEN_PROCESSING_PRESENTATION | |
|  | 0.000000101988173462075 | | REACTOME_SIGNALLING_BY_NGF | |
|  | 0.000000141370418926812 | | REACTOME_SRP_DEPENDENT_COTRANSLATIONAL_PROTEIN_TARGETING_TO_MEMBRANE | |
|  | 0.000000158760680601944 | | REACTOME_APOPTOSIS | |
|  | 0.000000172465027814539 | | KEGG_ECM_RECEPTOR_INTERACTION | |
|  | 0.000000213563295714536 | | REACTOME_DNA_REPAIR | |
