# Supplementary material for: Classifying Breast Cancer Subtypes Using Multiple Kernel Learning Based on Omics Data
Source: Genes (Basel). 2019 Mar 7;10(3):200. doi: 10.3390/genes10030200 (PMC6471546; doi:10.3390/genes10030200)
Supplement: Supplementary file 1 [file genes-10-00200-s001.zip › Table S4. The top 30 pathways in these classification on methylation data.docx]

Table S4. The top 30 pathways in these classification on methylation data

| **Classification** | **P-value** | | **PATHWAY** |
| --- | --- | --- | --- |
| **Luminal A**  **Versus**  **Luminal B** | 0.00000000000229272156815341 | | KEGG_NEUROACTIVE_LIGAND_RECEPTOR_INTERACTION |
|  | 0.00000000000405520061974585 | | REACTOME_NEURONAL_SYSTEM |
|  | 0.000000000546596545802913 | | REACTOME_GPCR_LIGAND_BINDING |
|  | 0.00000000914552500397292 | | REACTOME_FGFR1_LIGAND_BINDING_AND_ACTIVATION |
|  | 0.0000000366578440935683 | | REACTOME_ACTIVATED_POINT_MUTANTS_OF_FGFR2 |
|  | 0.000000115766098818249 | | KEGG_REGULATION_OF_ACTIN_CYTOSKELETON |
|  | 0.000000171490691536214 | | REACTOME_COLLAGEN_FORMATION |
|  | 0.000000395138455955291 | | REACTOME_TRANSMISSION_ACROSS_CHEMICAL_SYNAPSES |
|  | 0.000000745382086941859 | | REACTOME_FGFR_LIGAND_BINDING_AND_ACTIVATION |
|  | 0.000000749301491631549 | | REACTOME_EXTRACELLULAR_MATRIX_ORGANIZATION |
|  | 0.000000918605489896862 | | REACTOME_CLASS_A1_RHODOPSIN_LIKE_RECEPTORS |
|  | 0.00000318877444560606 | | REACTOME_SIGNALING_BY_FGFR_MUTANTS |
|  | 0.00000318877444560606 | | REACTOME_VOLTAGE_GATED_POTASSIUM_CHANNELS |
|  | 0.00000354581581751834 | | REACTOME_POTASSIUM_CHANNELS |
|  | 0.0000052248727929749 | | REACTOME_NEGATIVE_REGULATION_OF_FGFR_SIGNALING |
|  | 0.00000593222444178476 | | REACTOME_SHC_MEDIATED_CASCADE |
|  | 0.0000061404105956564 | | REACTOME_SIGNALING_BY_GPCR |
|  | 0.0000102718281179692 | | REACTOME_DEVELOPMENTAL_BIOLOGY |
|  | 0.0000185846163268666 | | REACTOME_PEPTIDE_LIGAND_BINDING_RECEPTORS |
|  | 0.0000231081258599275 | | REACTOME_PHOSPHOLIPASE_C_MEDIATED_CASCADE |
|  | 0.0000277046294904215 | | REACTOME_SIGNALING_BY_ACTIVATED_POINT_MUTANTS_OF_FGFR1 |
|  | 0.0000283898913736591 | | REACTOME_NEUROTRANSMITTER_RELEASE_CYCLE |
|  | 0.0000323951019236546 | | KEGG_BASAL_CELL_CARCINOMA |
|  | 0.0000372892918357737 | | REACTOME_G_ALPHA_I_SIGNALLING_EVENTS |
|  | 0.000044279770882949 | | REACTOME_FRS2_MEDIATED_CASCADE |
|  | 0.0000460284841949932 | | REACTOME_FGFR2C_LIGAND_BINDING_AND_ACTIVATION |
|  | 0.0000460284841949932 | | REACTOME_FGFR4_LIGAND_BINDING_AND_ACTIVATION |
|  | 0.0000724909776880223 | | REACTOME_SYNTHESIS_SECRETION_AND_INACTIVATION_OF_GIP |
|  | 0.0000759173265633128 | | REACTOME_SIGNALING_BY_FGFR1_MUTANTS |
|  | 0.0000901800719119716 | | REACTOME_INCRETIN_SYNTHESIS_SECRETION_AND_INACTIVATION |
| **Luminal A**  **Versus**  **HER2 (+)** | 0.000000026018597609756 | REACTOME_GPCR_DOWNSTREAM_SIGNALING | |
|  | 0.0000000426287538601855 | REACTOME_SIGNALING_BY_GPCR | |
|  | 0.0000128636621328226 | REACTOME_GPCR_LIGAND_BINDING | |
|  | 0.0000153738038942697 | KEGG_LEUKOCYTE_TRANSENDOTHELIAL_MIGRATION | |
|  | 0.0000638441910435494 | REACTOME_CLASS_A1_RHODOPSIN_LIKE_RECEPTORS | |
|  | 0.0000656841407663533 | KEGG_CELL_ADHESION_MOLECULES_CAMS | |
|  | 0.000086338704957778 | KEGG_TRYPTOPHAN_METABOLISM | |
|  | 0.000105102072182839 | REACTOME_DEGRADATION_OF_THE_EXTRACELLULAR_MATRIX | |
|  | 0.000116682766908571 | REACTOME_G_ALPHA_Q_SIGNALLING_EVENTS | |
|  | 0.000119975344765377 | REACTOME_GASTRIN_CREB_SIGNALLING_PATHWAY_VIA_PKC_AND_MAPK | |
|  | 0.000148551941714592 | REACTOME_ACTIVATION_OF_CHAPERONES_BY_ATF6_ALPHA | |
|  | 0.000149104188115423 | KEGG_NEUROACTIVE_LIGAND_RECEPTOR_INTERACTION | |
|  | 0.00015560693367167 | REACTOME_GPVI_MEDIATED_ACTIVATION_CASCADE | |
|  | 0.000237413300749889 | REACTOME_G_ALPHA_I_SIGNALLING_EVENTS | |
|  | 0.000247375696931118 | KEGG_TYPE_II_DIABETES_MELLITUS | |
|  | 0.00030829198417659 | REACTOME_INITIAL_TRIGGERING_OF_COMPLEMENT | |
|  | 0.000313026848786979 | KEGG_PRION_DISEASES | |
|  | 0.000347416061548089 | REACTOME_BIOLOGICAL_OXIDATIONS | |
|  | 0.000497128364573718 | REACTOME_GABA_B_RECEPTOR_ACTIVATION | |
|  | 0.000563787790302528 | REACTOME_PLATELET_CALCIUM_HOMEOSTASIS | |
|  | 0.000591951142132285 | KEGG_INOSITOL_PHOSPHATE_METABOLISM | |
|  | 0.000629454346826042 | REACTOME_ELEVATION_OF_CYTOSOLIC_CA2_LEVELS | |
|  | 0.000824273245837781 | KEGG_ACUTE_MYELOID_LEUKEMIA | |
|  | 0.000824391047895556 | REACTOME_G_PROTEIN_BETA_GAMMA_SIGNALLING | |
|  | 0.000862279565834956 | KEGG_ALDOSTERONE_REGULATED_SODIUM_REABSORPTION | |
|  | 0.000941720799562673 | REACTOME_DESTABILIZATION_OF_MRNA_BY_TRISTETRAPROLIN_TTP | |
|  | 0.000986955328451389 | REACTOME_CREATION_OF_C4_AND_C2_ACTIVATORS | |
|  | 0.00100753630391248 | KEGG_PHOSPHATIDYLINOSITOL_SIGNALING_SYSTEM | |
|  | 0.00107333421111222 | KEGG_CALCIUM_SIGNALING_PATHWAY | |
|  | 0.00109826323723894 | REACTOME_UNFOLDED_PROTEIN_RESPONSE | |
| **Luminal A**  **Versus**  **TNBC** | 0 | REACTOME_GPCR_LIGAND_BINDING | |
|  | 0.000000000000000555111512312578 | KEGG_NEUROACTIVE_LIGAND_RECEPTOR_INTERACTION | |
|  | 0.00000000000000077715611723761 | REACTOME_CLASS_A1_RHODOPSIN_LIKE_RECEPTORS | |
|  | 0.00000000000000333066907387547 | REACTOME_SIGNALING_BY_GPCR | |
|  | 0.0000000000000197619698383278 | REACTOME_GPCR_DOWNSTREAM_SIGNALING | |
|  | 0.0000000000196626048776238 | REACTOME_PEPTIDE_LIGAND_BINDING_RECEPTORS | |
|  | 0.000000000308420733396986 | KEGG_CYTOKINE_CYTOKINE_RECEPTOR_INTERACTION | |
|  | 0.00000000979953629354213 | REACTOME_NEURONAL_SYSTEM | |
|  | 0.0000000127606365474264 | REACTOME_G_ALPHA_I_SIGNALLING_EVENTS | |
|  | 0.000000183513167995031 | REACTOME_CELL_SURFACE_INTERACTIONS_AT_THE_VASCULAR_WALL | |
|  | 0.000000246899766187525 | KEGG_LEUKOCYTE_TRANSENDOTHELIAL_MIGRATION | |
|  | 0.00000053315886872074 | REACTOME_G_ALPHA_S_SIGNALLING_EVENTS | |
|  | 0.00000108548952226784 | REACTOME_POTASSIUM_CHANNELS | |
|  | 0.00000121359128235632 | REACTOME_G_ALPHA_Q_SIGNALLING_EVENTS | |
|  | 0.00000317074568823017 | REACTOME_TRANSMEMBRANE_TRANSPORT_OF_SMALL_MOLECULES | |
|  | 0.00000575771741451181 | REACTOME_HEMOSTASIS | |
|  | 0.00000619014029790144 | REACTOME_PHASE1_FUNCTIONALIZATION_OF_COMPOUNDS | |
|  | 0.0000103644144704429 | KEGG_CHEMOKINE_SIGNALING_PATHWAY | |
|  | 0.0000125306123311963 | REACTOME_VOLTAGE_GATED_POTASSIUM_CHANNELS | |
|  | 0.0000143746246648035 | KEGG_CELL_ADHESION_MOLECULES_CAMS | |
|  | 0.0000174784697740682 | REACTOME_GASTRIN_CREB_SIGNALLING_PATHWAY_VIA_PKC_AND_MAPK | |
|  | 0.0000308151780751276 | REACTOME_TRANSMISSION_ACROSS_CHEMICAL_SYNAPSES | |
|  | 0.0000372049691629295 | KEGG_CALCIUM_SIGNALING_PATHWAY | |
|  | 0.0000404913064480095 | REACTOME_O_LINKED_GLYCOSYLATION_OF_MUCINS | |
|  | 0.0000685450303675728 | REACTOME_BIOLOGICAL_OXIDATIONS | |
|  | 0.0000762565205884025 | REACTOME_ADENYLATE_CYCLASE_ACTIVATING_PATHWAY | |
|  | 0.0000762565205884025 | REACTOME_NOREPINEPHRINE_NEUROTRANSMITTER_RELEASE_CYCLE | |
|  | 0.0000864685284057698 | REACTOME_COMMON_PATHWAY | |
|  | 0.000105210127818722 | REACTOME_REGULATION_OF_INSULIN_SECRETION | |
|  | 0.000121070285264535 | REACTOME_NEUROTRANSMITTER_RECEPTOR_BINDING_AND_DOWNSTREAM_TRANSMISSION_IN_THE_POSTSYNAPTIC_CELL | |
| **Luminal B**  **Versus**  **HER2 (+)** | 0.0000255566885972724 | REACTOME_NUCLEAR_RECEPTOR_TRANSCRIPTION_PATHWAY | |
|  | 0.0000815072209158219 | KEGG_HEDGEHOG_SIGNALING_PATHWAY | |
|  | 0.000171582288242766 | REACTOME_CLASS_A1_RHODOPSIN_LIKE_RECEPTORS | |
|  | 0.000185218561590705 | REACTOME_DEGRADATION_OF_THE_EXTRACELLULAR_MATRIX | |
|  | 0.00021085020409839 | REACTOME_GPCR_LIGAND_BINDING | |
|  | 0.00025695827360972 | REACTOME_INWARDLY_RECTIFYING_K_CHANNELS | |
|  | 0.000289403348442518 | REACTOME_G_ALPHA_I_SIGNALLING_EVENTS | |
|  | 0.000369950947307074 | REACTOME_HEPARAN_SULFATE_HEPARIN_HS_GAG_METABOLISM | |
|  | 0.000401810981511708 | KEGG_FRUCTOSE_AND_MANNOSE_METABOLISM | |
|  | 0.000438253387208709 | REACTOME_GASTRIN_CREB_SIGNALLING_PATHWAY_VIA_PKC_AND_MAPK | |
|  | 0.000618168312733092 | REACTOME_G_ALPHA_Q_SIGNALLING_EVENTS | |
|  | 0.000681589870179855 | REACTOME_GABA_B_RECEPTOR_ACTIVATION | |
|  | 0.000710793257793885 | REACTOME_REGULATION_OF_INSULIN_SECRETION_BY_ACETYLCHOLINE | |
|  | 0.000781747727318538 | REACTOME_GENERIC_TRANSCRIPTION_PATHWAY | |
|  | 0.000926333083210329 | REACTOME_GLYCOSAMINOGLYCAN_METABOLISM | |
|  | 0.00096377584201901 | REACTOME_ACTIVATION_OF_CHAPERONES_BY_ATF6_ALPHA | |
|  | 0.00108930509347005 | KEGG_TGF_BETA_SIGNALING_PATHWAY | |
|  | 0.00112618610790927 | REACTOME_A_TETRASACCHARIDE_LINKER_SEQUENCE_IS_REQUIRED_FOR_GAG_SYNTHESIS | |
|  | 0.00112618610790927 | REACTOME_INHIBITION_OF_VOLTAGE_GATED_CA2_CHANNELS_VIA_GBETA_GAMMA_SUBUNITS | |
|  | 0.00121060325650368 | REACTOME_REGULATION_OF_WATER_BALANCE_BY_RENAL_AQUAPORINS | |
|  | 0.00126721581027656 | REACTOME_P2Y_RECEPTORS | |
|  | 0.00126721581027656 | REACTOME_TANDEM_PORE_DOMAIN_POTASSIUM_CHANNELS | |
|  | 0.00151689655729514 | REACTOME_GENERATION_OF_SECOND_MESSENGER_MOLECULES | |
|  | 0.00156083918983918 | KEGG_LEUKOCYTE_TRANSENDOTHELIAL_MIGRATION | |
|  | 0.00162455917665705 | REACTOME_RETROGRADE_NEUROTROPHIN_SIGNALLING | |
|  | 0.00162455917665705 | REACTOME_INITIAL_TRIGGERING_OF_COMPLEMENT | |
|  | 0.00174376805527787 | REACTOME_MYOGENESIS | |
|  | 0.00181456766459009 | KEGG_NOTCH_SIGNALING_PATHWAY | |
|  | 0.00181877868894043 | REACTOME_SIGNALING_BY_GPCR | |
|  | 0.0020390050271285 | REACTOME_HYALURONAN_METABOLISM | |
| **Luminal B**  **Versus**  **TNBC** | 0 | KEGG_NEUROACTIVE_LIGAND_RECEPTOR_INTERACTION | |
|  | 0 | REACTOME_GPCR_LIGAND_BINDING | |
|  | 0.000000000000000111022302462516 | REACTOME_SIGNALING_BY_GPCR | |
|  | 0.000000000000403788114056169 | REACTOME_GPCR_DOWNSTREAM_SIGNALING | |
|  | 0.0000000000032692737406137 | REACTOME_CLASS_A1_RHODOPSIN_LIKE_RECEPTORS | |
|  | 0.0000000000145148337793444 | REACTOME_NEURONAL_SYSTEM | |
|  | 0.00000000990380477627895 | REACTOME_PEPTIDE_LIGAND_BINDING_RECEPTORS | |
|  | 0.0000000109965456740468 | KEGG_LEUKOCYTE_TRANSENDOTHELIAL_MIGRATION | |
|  | 0.0000000896030156738448 | REACTOME_POTASSIUM_CHANNELS | |
|  | 0.000000196401960805836 | KEGG_CYTOKINE_CYTOKINE_RECEPTOR_INTERACTION | |
|  | 0.000000332901379573869 | REACTOME_HEMOSTASIS | |
|  | 0.00000035401357201259 | REACTOME_G_ALPHA_I_SIGNALLING_EVENTS | |
|  | 0.00000038787894884873 | REACTOME_CELL_SURFACE_INTERACTIONS_AT_THE_VASCULAR_WALL | |
|  | 0.00000075462290549666 | REACTOME_PLATELET_ACTIVATION_SIGNALING_AND_AGGREGATION | |
|  | 0.00000097313945102151 | REACTOME_TRANSMISSION_ACROSS_CHEMICAL_SYNAPSES | |
|  | 0.00000133182444672642 | KEGG_CALCIUM_SIGNALING_PATHWAY | |
|  | 0.00000136366346259464 | KEGG_HEDGEHOG_SIGNALING_PATHWAY | |
|  | 0.00000300357980953514 | REACTOME_CLASS_B_2_SECRETIN_FAMILY_RECEPTORS | |
|  | 0.00000548961053792318 | KEGG_BASAL_CELL_CARCINOMA | |
|  | 0.0000055122122845086 | REACTOME_G_ALPHA_Q_SIGNALLING_EVENTS | |
|  | 0.00000586439086780466 | REACTOME_PLATELET_ADHESION_TO_EXPOSED_COLLAGEN | |
|  | 0.0000104418323412236 | REACTOME_NEUROTRANSMITTER_RECEPTOR_BINDING_AND_DOWNSTREAM_TRANSMISSION_IN_THE_POSTSYNAPTIC_CELL | |
|  | 0.0000126385739451784 | REACTOME_TRANSMEMBRANE_TRANSPORT_OF_SMALL_MOLECULES | |
|  | 0.000015198413447437 | KEGG_CHEMOKINE_SIGNALING_PATHWAY | |
|  | 0.0000174803327659401 | KEGG_JAK_STAT_SIGNALING_PATHWAY | |
|  | 0.0000249159550721467 | REACTOME_SLC_MEDIATED_TRANSMEMBRANE_TRANSPORT | |
|  | 0.0000367402034782138 | REACTOME_VOLTAGE_GATED_POTASSIUM_CHANNELS | |
|  | 0.0000404712202060065 | KEGG_FC_EPSILON_RI_SIGNALING_PATHWAY | |
|  | 0.0000414081231248087 | REACTOME_GPVI_MEDIATED_ACTIVATION_CASCADE | |
|  | 0.0000423664198226925 | KEGG_APOPTOSIS | |
| **HER2 (+)**  **Versus**  **TNBC** | 0.00000000185437454280901 | REACTOME_SIGNALING_BY_GPCR | |
|  | 0.00000000460634053123243 | REACTOME_GPCR_DOWNSTREAM_SIGNALING | |
|  | 0.000000100869273600424 | KEGG_NEUROACTIVE_LIGAND_RECEPTOR_INTERACTION | |
|  | 0.000000243103027597336 | REACTOME_GPCR_LIGAND_BINDING | |
|  | 0.00000234454796532724 | REACTOME_GABA_RECEPTOR_ACTIVATION | |
|  | 0.00000273529492023084 | REACTOME_G_ALPHA_I_SIGNALLING_EVENTS | |
|  | 0.00000628009227487958 | REACTOME_TRANSMISSION_ACROSS_CHEMICAL_SYNAPSES | |
|  | 0.00000767941520529725 | REACTOME_NEURONAL_SYSTEM | |
|  | 0.0000151542098657531 | REACTOME_NEUROTRANSMITTER_RECEPTOR_BINDING_AND_DOWNSTREAM_TRANSMISSION_IN_THE_POSTSYNAPTIC_CELL | |
|  | 0.0000236121911556531 | REACTOME_GABA_B_RECEPTOR_ACTIVATION | |
|  | 0.0000297171756867831 | REACTOME_STRIATED_MUSCLE_CONTRACTION | |
|  | 0.0000538712585013146 | REACTOME_ADENYLATE_CYCLASE_ACTIVATING_PATHWAY | |
|  | 0.0000538712585013146 | REACTOME_NOREPINEPHRINE_NEUROTRANSMITTER_RELEASE_CYCLE | |
|  | 0.0000744163551212873 | REACTOME_CLASS_A1_RHODOPSIN_LIKE_RECEPTORS | |
|  | 0.0000952675072595222 | REACTOME_TRANSMEMBRANE_TRANSPORT_OF_SMALL_MOLECULES | |
|  | 0.000173507957454411 | REACTOME_ADENYLATE_CYCLASE_INHIBITORY_PATHWAY | |
|  | 0.000238461700365233 | REACTOME_CLASS_C_3_METABOTROPIC_GLUTAMATE_PHEROMONE_RECEPTORS | |
|  | 0.000238943202554465 | KEGG_MATURITY_ONSET_DIABETES_OF_THE_YOUNG | |
|  | 0.000302567021925992 | REACTOME_CELL_CELL_JUNCTION_ORGANIZATION | |
|  | 0.000366589606320633 | REACTOME_PEPTIDE_LIGAND_BINDING_RECEPTORS | |
|  | 0.000405546637401222 | REACTOME_INTEGRATION_OF_ENERGY_METABOLISM | |
|  | 0.00049472054769073 | REACTOME_TIGHT_JUNCTION_INTERACTIONS | |
|  | 0.000496124384293695 | REACTOME_G_ALPHA_S_SIGNALLING_EVENTS | |
|  | 0.000498767685097379 | KEGG_CHEMOKINE_SIGNALING_PATHWAY | |
|  | 0.000536430650743824 | REACTOME_PKA_MEDIATED_PHOSPHORYLATION_OF_CREB | |
|  | 0.000536430650743824 | REACTOME_NA_CL_DEPENDENT_NEUROTRANSMITTER_TRANSPORTERS | |
|  | 0.000582427418226783 | REACTOME_REGULATION_OF_BETA_CELL_DEVELOPMENT | |
|  | 0.000647358206230741 | REACTOME_MUSCLE_CONTRACTION | |
|  | 0.000681295850805452 | REACTOME_INWARDLY_RECTIFYING_K_CHANNELS | |
|  | 0.00072771836690011 | REACTOME_NUCLEAR_RECEPTOR_TRANSCRIPTION_PATHWAY | |
