# Supplementary material for: Classifying Breast Cancer Subtypes Using Multiple Kernel Learning Based on Omics Data
Source: Genes (Basel). 2019 Mar 7;10(3):200. doi: 10.3390/genes10030200 (PMC6471546; doi:10.3390/genes10030200)
Supplement: Supplementary file 1 [file genes-10-00200-s001.zip › Table S5. The top 30 pathways in these classification on CNV data.docx]

Table S5. The top 30 pathways in these classification on CNV data

| **Classification** | **P-value** | **PATHWAY** |  |
| --- | --- | --- | --- |
| **Luminal A**  **Versus**  **Luminal B** | 0.000000000957702472881294 | KEGG_ARACHIDONIC_ACID_METABOLISM |  |
|  | 0.00000000165381219829897 | KEGG_PATHWAYS_IN_CANCER |  |
|  | 0.00000000281157752635153 | REACTOME_IMMUNE_SYSTEM |  |
|  | 0.00000000368522079519096 | KEGG_INSULIN_SIGNALING_PATHWAY |  |
|  | 0.00000000994056426062428 | REACTOME_METABOLISM_OF_LIPIDS_AND_LIPOPROTEINS |  |
|  | 0.0000000177670558265675 | REACTOME_CYTOKINE_SIGNALING_IN_IMMUNE_SYSTEM |  |
|  | 0.0000000323095279419761 | KEGG_MAPK_SIGNALING_PATHWAY |  |
|  | 0.0000000413080742944771 | REACTOME_SIGNALING_BY_ILS |  |
|  | 0.000000107181871378259 | KEGG_ACUTE_MYELOID_LEUKEMIA |  |
|  | 0.000000135419106372936 | KEGG_VEGF_SIGNALING_PATHWAY |  |
|  | 0.000000178592969857228 | KEGG_NON_SMALL_CELL_LUNG_CANCER |  |
|  | 0.000000229163383269615 | KEGG_CHRONIC_MYELOID_LEUKEMIA |  |
|  | 0.000000334181316619109 | REACTOME_DEVELOPMENTAL_BIOLOGY |  |
|  | 0.000000887759562195889 | KEGG_LINOLEIC_ACID_METABOLISM |  |
|  | 0.00000111457241047663 | KEGG_VASCULAR_SMOOTH_MUSCLE_CONTRACTION |  |
|  | 0.00000114016377417148 | REACTOME_PHOSPHOLIPID_METABOLISM |  |
|  | 0.00000124405730950183 | KEGG_FC_EPSILON_RI_SIGNALING_PATHWAY |  |
|  | 0.00000130794970742976 | KEGG_ALPHA_LINOLENIC_ACID_METABOLISM |  |
|  | 0.00000157794103061715 | KEGG_PANCREATIC_CANCER |  |
|  | 0.00000161386615382675 | REACTOME_SLC_MEDIATED_TRANSMEMBRANE_TRANSPORT |  |
|  | 0.00000164587403173044 | REACTOME_TRANSMEMBRANE_TRANSPORT_OF_SMALL_MOLECULES |  |
|  | 0.00000173846578555015 | KEGG_GNRH_SIGNALING_PATHWAY |  |
|  | 0.00000174865755475651 | REACTOME_METABOLISM_OF_PROTEINS |  |
|  | 0.00000189885515888832 | REACTOME_LYSOSOME_VESICLE_BIOGENESIS |  |
|  | 0.00000277717787600107 | KEGG_CALCIUM_SIGNALING_PATHWAY |  |
|  | 0.00000507056261456196 | REACTOME_NGF_SIGNALLING_VIA_TRKA_FROM_THE_PLASMA_MEMBRANE |  |
|  | 0.00000590706660830165 | KEGG_HUNTINGTONS_DISEASE |  |
|  | 0.0000059405034744664 | REACTOME_SIGNALING_BY_SCF_KIT |  |
|  | 0.00000599707213022604 | KEGG_GLIOMA |  |
|  | 0.00000864846881110104 | KEGG_APOPTOSIS |  |
| **Luminal A**  **Versus**  **HER2 (+)** | 0 | REACTOME_GENERIC_TRANSCRIPTION_PATHWAY | |
|  | 0 | REACTOME_RNA_POL_I_PROMOTER_OPENING | |
|  | 0.000000000000000111022302462516 | REACTOME_MEIOSIS | |
|  | 0.000000000000000111022302462516 | REACTOME_RNA_POL_I_TRANSCRIPTION | |
|  | 0.000000000000000333066907387547 | REACTOME_PACKAGING_OF_TELOMERE_ENDS | |
|  | 0.000000000000000888178419700125 | REACTOME_MEIOTIC_RECOMBINATION | |
|  | 0.00000000000000177635683940025 | REACTOME_MEIOTIC_SYNAPSIS | |
|  | 0.00000000000000599520433297585 | REACTOME_AMYLOIDS | |
|  | 0.0000000000000288657986402541 | REACTOME_CHROMOSOME_MAINTENANCE | |
|  | 0.0000000000000440758540776187 | REACTOME_DEPOSITION_OF_NEW_CENPA_CONTAINING_NUCLEOSOMES_AT_THE_CENTROMERE | |
|  | 0.000000000000104027897407377 | REACTOME_TELOMERE_MAINTENANCE | |
|  | 0.0000000000037327918533947 | REACTOME_RNA_POL_I_RNA_POL_III_AND_MITOCHONDRIAL_TRANSCRIPTION | |
|  | 0.0000000000140799594205987 | REACTOME_IMMUNE_SYSTEM | |
|  | 0.0000000000462804239376169 | REACTOME_TRANSCRIPTION | |
|  | 0.000000000067236438638929 | REACTOME_CELL_CYCLE | |
|  | 0.0000000000836536395709686 | KEGG_SYSTEMIC_LUPUS_ERYTHEMATOSUS | |
|  | 0.000000012551402250871 | REACTOME_TRANSMEMBRANE_TRANSPORT_OF_SMALL_MOLECULES | |
|  | 0.0000000144227270126862 | REACTOME_SIGNALLING_BY_NGF | |
|  | 0.0000000284702601494047 | REACTOME_DOWNSTREAM_SIGNAL_TRANSDUCTION | |
|  | 0.000000029984135840877 | REACTOME_SLC_MEDIATED_TRANSMEMBRANE_TRANSPORT | |
|  | 0.0000000408656746220259 | REACTOME_METABOLISM_OF_LIPIDS_AND_LIPOPROTEINS | |
|  | 0.0000000946375786625708 | REACTOME_MRNA_PROCESSING | |
|  | 0.000000095675052325106 | REACTOME_ADAPTIVE_IMMUNE_SYSTEM | |
|  | 0.000000126346650142217 | REACTOME_METABOLISM_OF_PROTEINS | |
|  | 0.000000205248478657438 | REACTOME_METABOLISM_OF_RNA | |
|  | 0.000000331549408261189 | KEGG_JAK_STAT_SIGNALING_PATHWAY | |
|  | 0.000000510661173125371 | REACTOME_SIGNALING_BY_ERBB2 | |
|  | 0.000000680804067676455 | REACTOME_TRANSPORT_OF_INORGANIC_CATIONS_ANIONS_AND_AMINO_ACIDS_OLIGOPEPTIDES | |
|  | 0.000000728211335343509 | REACTOME_NEURONAL_SYSTEM | |
|  | 0.000000749284588597021 | REACTOME_TRANSMISSION_ACROSS_CHEMICAL_SYNAPSES | |
| **Luminal A**  **Versus**  **TNBC** | 0 | KEGG_JAK_STAT_SIGNALING_PATHWAY |  |
|  | 0 | REACTOME_DEVELOPMENTAL_BIOLOGY |  |
|  | 0 | REACTOME_CELL_CYCLE |  |
|  | 0 | REACTOME_NEURONAL_SYSTEM |  |
|  | 0 | REACTOME_SIGNALING_BY_GPCR |  |
|  | 0 | REACTOME_CELL_CYCLE_MITOTIC |  |
|  | 0 | REACTOME_TRANSMEMBRANE_TRANSPORT_OF_SMALL_MOLECULES |  |
|  | 0 | REACTOME_METABOLISM_OF_PROTEINS |  |
|  | 0 | REACTOME_AXON_GUIDANCE |  |
|  | 0 | REACTOME_SLC_MEDIATED_TRANSMEMBRANE_TRANSPORT |  |
|  | 0 | REACTOME_GPCR_DOWNSTREAM_SIGNALING |  |
|  | 0 | REACTOME_METABOLISM_OF_LIPIDS_AND_LIPOPROTEINS |  |
|  | 0 | REACTOME_GABA_A_RECEPTOR_ACTIVATION |  |
|  | 0 | REACTOME_HEMOSTASIS |  |
|  | 0 | REACTOME_IMMUNE_SYSTEM |  |
|  | 0 | REACTOME_ADAPTIVE_IMMUNE_SYSTEM |  |
|  | 0 | REACTOME_CYTOKINE_SIGNALING_IN_IMMUNE_SYSTEM |  |
|  | 0 | REACTOME_ANTIGEN_PROCESSING_UBIQUITINATION_PROTEASOME_DEGRADATION |  |
|  | 0.000000000000000111022302462516 | REACTOME_METABOLISM_OF_CARBOHYDRATES |  |
|  | 0.000000000000000111022302462516 | REACTOME_CLASS_I_MHC_MEDIATED_ANTIGEN_PROCESSING_PRESENTATION |  |
|  | 0.000000000000000888178419700125 | REACTOME_TRANSMISSION_ACROSS_CHEMICAL_SYNAPSES |  |
|  | 0.00000000000000133226762955019 | REACTOME_METABOLISM_OF_RNA |  |
|  | 0.00000000000000255351295663786 | KEGG_NEUROACTIVE_LIGAND_RECEPTOR_INTERACTION |  |
|  | 0.00000000000000266453525910038 | REACTOME_FATTY_ACID_TRIACYLGLYCEROL_AND_KETONE_BODY_METABOLISM |  |
|  | 0.00000000000000321964677141295 | REACTOME_PLATELET_ACTIVATION_SIGNALING_AND_AGGREGATION |  |
|  | 0.000000000000007105427357601 | REACTOME_INTERFERON_SIGNALING |  |
|  | 0.0000000000000418554080283684 | KEGG_PATHWAYS_IN_CANCER |  |
|  | 0.000000000000126343380202343 | REACTOME_HIV_INFECTION |  |
|  | 0.000000000000208721928629529 | KEGG_MAPK_SIGNALING_PATHWAY |  |
|  | 0.0000000000002647881913731 | REACTOME_NEUROTRANSMITTER_RECEPTOR_BINDING_AND_DOWNSTREAM_TRANSMISSION_IN_THE_POSTSYNAPTIC_CELL |  |
| **Luminal B**  **Versus**  **ERBB2** | 0 | KEGG_SYSTEMIC_LUPUS_ERYTHEMATOSUS |  |
|  | 0 | REACTOME_MEIOSIS |  |
|  | 0 | REACTOME_RNA_POL_I_TRANSCRIPTION |  |
|  | 0 | REACTOME_TRANSCRIPTION |  |
|  | 0 | REACTOME_RNA_POL_I_RNA_POL_III_AND_MITOCHONDRIAL_TRANSCRIPTION |  |
|  | 0 | REACTOME_CHROMOSOME_MAINTENANCE |  |
|  | 0 | REACTOME_DEPOSITION_OF_NEW_CENPA_CONTAINING_NUCLEOSOMES_AT_THE_CENTROMERE |  |
|  | 0 | REACTOME_RNA_POL_I_PROMOTER_OPENING |  |
|  | 0 | REACTOME_MEIOTIC_RECOMBINATION |  |
|  | 0 | REACTOME_MEIOTIC_SYNAPSIS |  |
|  | 0 | REACTOME_AMYLOIDS |  |
|  | 0 | REACTOME_PACKAGING_OF_TELOMERE_ENDS |  |
|  | 0 | REACTOME_TELOMERE_MAINTENANCE |  |
|  | 0.0000000000000721644966006352 | REACTOME_CELL_CYCLE |  |
|  | 0.000000107940427485076 | REACTOME_CELL_CELL_JUNCTION_ORGANIZATION |  |
|  | 0.00000103083577096541 | REACTOME_ADHERENS_JUNCTIONS_INTERACTIONS |  |
|  | 0.00000392984186892154 | REACTOME_CELL_JUNCTION_ORGANIZATION |  |
|  | 0.00000641616085172991 | KEGG_ABC_TRANSPORTERS |  |
|  | 0.000016638846938033 | KEGG_WNT_SIGNALING_PATHWAY |  |
|  | 0.0000220673697687301 | REACTOME_DEGRADATION_OF_THE_EXTRACELLULAR_MATRIX |  |
|  | 0.0000542582305858019 | REACTOME_ABC_FAMILY_PROTEINS_MEDIATED_TRANSPORT |  |
|  | 0.0000722794014812456 | REACTOME_CELL_CELL_COMMUNICATION |  |
|  | 0.0000897046321788331 | REACTOME_INHIBITION_OF_VOLTAGE_GATED_CA2_CHANNELS_VIA_GBETA_GAMMA_SUBUNITS |  |
|  | 0.000124218096369089 | REACTOME_ABCA_TRANSPORTERS_IN_LIPID_HOMEOSTASIS |  |
|  | 0.000140215913108976 | REACTOME_GABA_B_RECEPTOR_ACTIVATION |  |
|  | 0.000141671209168415 | REACTOME_FACTORS_INVOLVED_IN_MEGAKARYOCYTE_DEVELOPMENT_AND_PLATELET_PRODUCTION |  |
|  | 0.000142261371302688 | REACTOME_TRAFFICKING_OF_AMPA_RECEPTORS |  |
|  | 0.000163379680991893 | REACTOME_HEMOSTASIS |  |
|  | 0.000246535075062893 | KEGG_LEUKOCYTE_TRANSENDOTHELIAL_MIGRATION |  |
|  | 0.000262107987375026 | REACTOME_NEUROTRANSMITTER_RECEPTOR_BINDING_AND_DOWNSTREAM_TRANSMISSION_IN_THE_POSTSYNAPTIC_CELL |  |
| **Luminal B**  **Versus**  **TNBC** | 0 | KEGG_JAK_STAT_SIGNALING_PATHWAY |  |
|  | 0 | REACTOME_DEVELOPMENTAL_BIOLOGY |  |
|  | 0 | REACTOME_TETRAHYDROBIOPTERIN_BH4_SYNTHESIS_RECYCLING_SALVAGE_AND_REGULATION |  |
|  | 0 | REACTOME_CELL_CYCLE |  |
|  | 0 | REACTOME_NEURONAL_SYSTEM |  |
|  | 0 | REACTOME_CELL_CYCLE_MITOTIC |  |
|  | 0 | REACTOME_TRANSMEMBRANE_TRANSPORT_OF_SMALL_MOLECULES |  |
|  | 0 | REACTOME_HEMOSTASIS |  |
|  | 0 | REACTOME_IMMUNE_SYSTEM |  |
|  | 0 | REACTOME_ADAPTIVE_IMMUNE_SYSTEM |  |
|  | 0.000000000000000111022302462516 | REACTOME_METABOLISM_OF_LIPIDS_AND_LIPOPROTEINS |  |
|  | 0.000000000000000222044604925031 | KEGG_NEUROACTIVE_LIGAND_RECEPTOR_INTERACTION |  |
|  | 0.000000000000000555111512312578 | REACTOME_METABOLISM_OF_PROTEINS |  |
|  | 0.00000000000000310862446895044 | REACTOME_SLC_MEDIATED_TRANSMEMBRANE_TRANSPORT |  |
|  | 0.0000000000000214273043752655 | REACTOME_SIGNALING_BY_GPCR |  |
|  | 0.0000000000000559552404411079 | REACTOME_GPCR_DOWNSTREAM_SIGNALING |  |
|  | 0.000000000000678346268045971 | REACTOME_AXON_GUIDANCE |  |
|  | 0.000000000000891064999564151 | REACTOME_TRANSMISSION_ACROSS_CHEMICAL_SYNAPSES |  |
|  | 0.00000000000273270295281236 | REACTOME_ANTIGEN_PROCESSING_UBIQUITINATION_PROTEASOME_DEGRADATION |  |
|  | 0.00000000000950728384907507 | REACTOME_CLASS_I_MHC_MEDIATED_ANTIGEN_PROCESSING_PRESENTATION |  |
|  | 0.0000000000127270416427905 | REACTOME_SIGNALING_BY_THE_B_CELL_RECEPTOR_BCR |  |
|  | 0.0000000000161218816074893 | KEGG_CALCIUM_SIGNALING_PATHWAY |  |
|  | 0.0000000000181531456533435 | REACTOME_METABOLISM_OF_AMINO_ACIDS_AND_DERIVATIVES |  |
|  | 0.0000000000237171393635549 | REACTOME_METABOLISM_OF_CARBOHYDRATES |  |
|  | 0.0000000000429016822067751 | REACTOME_MITOTIC_G1_G1_S_PHASES |  |
|  | 0.0000000000574023051314043 | REACTOME_CYTOKINE_SIGNALING_IN_IMMUNE_SYSTEM |  |
|  | 0.0000000000634859942394428 | REACTOME_INNATE_IMMUNE_SYSTEM |  |
|  | 0.000000000083504425596459 | REACTOME_NEUROTRANSMITTER_RECEPTOR_BINDING_AND_DOWNSTREAM_TRANSMISSION_IN_THE_POSTSYNAPTIC_CELL |  |
|  | 0.0000000000855017168177596 | REACTOME_FATTY_ACID_TRIACYLGLYCEROL_AND_KETONE_BODY_METABOLISM |  |
|  | 0.0000000000909474717758485 | REACTOME_MEIOSIS |  |
| **HER2 (+)**  **Versus**  **TNBC** | 0 | REACTOME_GENERIC_TRANSCRIPTION_PATHWAY |  |
|  | 0.0000000364564868293726 | REACTOME_INTEGRIN_CELL_SURFACE_INTERACTIONS |  |
|  | 0.000000300453246393495 | KEGG_REGULATION_OF_ACTIN_CYTOSKELETON |  |
|  | 0.000000675656482873599 | REACTOME_HEMOSTASIS |  |
|  | 0.00000136918711812761 | KEGG_FOCAL_ADHESION |  |
|  | 0.00000137685603918136 | REACTOME_SIGNALING_BY_PDGF |  |
|  | 0.00000209676252305169 | REACTOME_DEVELOPMENTAL_BIOLOGY |  |
|  | 0.00000268383206070144 | REACTOME_MITOTIC_G1_G1_S_PHASES |  |
|  | 0.00000334320565253954 | REACTOME_PLATELET_ACTIVATION_SIGNALING_AND_AGGREGATION |  |
|  | 0.00000355566521126338 | REACTOME_METABOLISM_OF_PROTEINS |  |
|  | 0.00000684618606983101 | REACTOME_METABOLISM_OF_LIPIDS_AND_LIPOPROTEINS |  |
|  | 0.00000755338838864805 | REACTOME_ADAPTIVE_IMMUNE_SYSTEM |  |
|  | 0.00000873106667254042 | REACTOME_IMMUNE_SYSTEM |  |
|  | 0.00000936369121729008 | REACTOME_POST_TRANSLATIONAL_PROTEIN_MODIFICATION |  |
|  | 0.00000982531312032631 | REACTOME_METABOLISM_OF_RNA |  |
|  | 0.0000111190630937008 | REACTOME_TRANSMEMBRANE_TRANSPORT_OF_SMALL_MOLECULES |  |
|  | 0.0000123538111957977 | REACTOME_PERK_REGULATED_GENE_EXPRESSION |  |
|  | 0.000024305300161509 | REACTOME_CLASS_I_MHC_MEDIATED_ANTIGEN_PROCESSING_PRESENTATION |  |
|  | 0.000025334711746261 | REACTOME_METABOLISM_OF_MRNA |  |
|  | 0.0000274216614696288 | REACTOME_G1_PHASE |  |
|  | 0.0000301935625326433 | REACTOME_ACTIVATION_OF_GENES_BY_ATF4 |  |
|  | 0.0000339045742551169 | KEGG_ECM_RECEPTOR_INTERACTION |  |
|  | 0.0000339045742551169 | KEGG_SMALL_CELL_LUNG_CANCER |  |
|  | 0.0000423493726063873 | REACTOME_CELL_CYCLE_MITOTIC |  |
|  | 0.0000466350855078401 | REACTOME_METABOLISM_OF_AMINO_ACIDS_AND_DERIVATIVES |  |
|  | 0.000047751311969324 | KEGG_MELANOMA |  |
|  | 0.0000494138884263862 | KEGG_GLYCINE_SERINE_AND_THREONINE_METABOLISM |  |
|  | 0.0000626002099568534 | REACTOME_ANTIGEN_PROCESSING_UBIQUITINATION_PROTEASOME_DEGRADATION |  |
|  | 0.0000666435172780311 | REACTOME_NEURONAL_SYSTEM |  |
|  | 0.0000732258413507214 | REACTOME_RESPONSE_TO_ELEVATED_PLATELET_CYTOSOLIC_CA2_ |  |
