# Supplementary figures and images for: Classifying Breast Cancer Subtypes Using Multiple Kernel Learning Based on Omics Data
Source: Genes (Basel). 2019 Mar 7;10(3):200. doi: 10.3390/genes10030200 (PMC6471546; doi:10.3390/genes10030200)

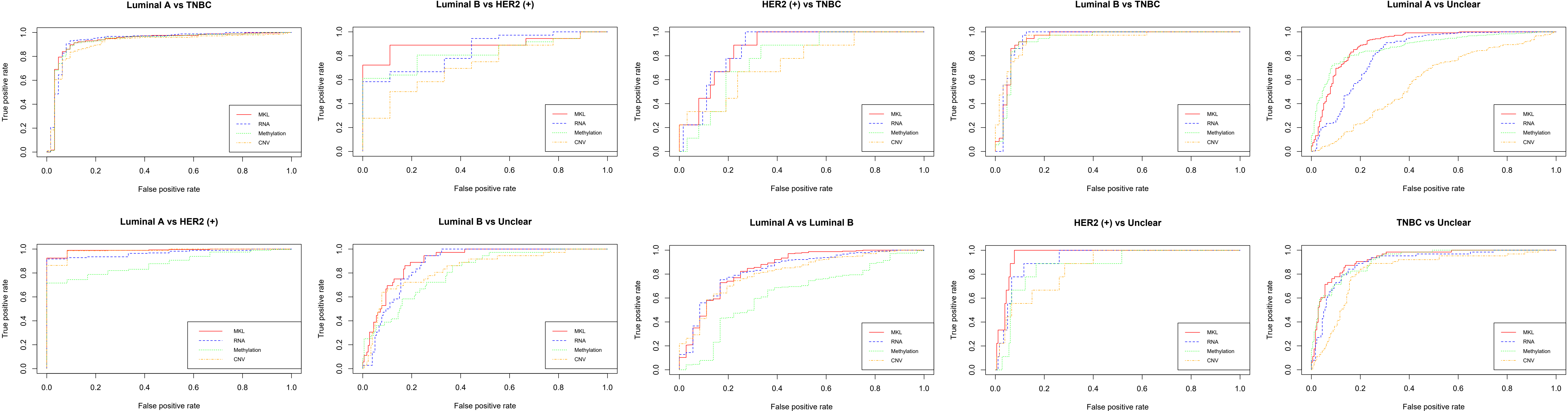

Supplement: Supplementary file 1 [file genes-10-00200-s001.zip › Figure S1. The ROC of any two breast cancer subtypes classification.pdf]
